# A Remarkable Case of a Gun-Shot Wound

**Published:** 1786

**Authors:** Barnabas Binney

**Affiliations:** Hospital Physician, and Surgeon in the American Army


IX. *A remarkable Caſe of a Gun-ſhot Wound. Communicated in a Letter from* Barnabas Binney, *Hoſpital Phyſician, and Surgeon in the American*

*American Army, in* 1782, *to the Honourable* Benjamin Lincoln, *Esq. F. A. A.*—From the *Memoirs of the American Academy of Arts and Sciences*, Vol. I. 4to. Boston, 1785.

ON April 9, 1782, David Beveridge, a seaman belonging to the sloop of war General Monk, was brought into the military hospital at this place, having been wounded the day before. He was a lad of about nineteen years of age, and in a good state of health, at the time of the action between the said ship and the Hyder Ally. In that action he was in the maintop of the Monk, when he received a musket ball in his belly from one of the marines on the quarter deck of the Hyder Ally, then within fifteen yards of the Monk. The ball entered his belly about two inches above his left groin, and within an inch of the anterior edge of the left ilium, passing out two inches on the right of the spine, between the two inferior true ribs, just touching the cartilage of the inferior angle of the right scapula. When he came into the hospital he had bled much, was very weak and cold, had a faultering voice, a cadaverous countenance, and a constant hiccup, while his fæces passed freely out of the wound in his belly. In this deplorable rable condition, in which neither art nor nature could promise any permanent relief, the only dictate of humanity was to smooth the path of death. As he was in great pain, I advised him to take a glass of Madeira wine, with twenty or thirty drops of laudanum in it, as often as necessary. He accordingly began, and continued this practice till the 13th, finding constant relief from it. He took no kind of sustenance all this time, excepting wine whey, never having any kind of discharge *ab ano* from the moment he was wounded, but constantly squirting with considerable force what fæces he had through the wound in his belly. On the 14th he had a common clyster administered, the greatest part of which also came out at the wound, the remainder coming as it went, *ab ano*, without bringing any fæces. From the 14th to the 18th he took considerable quantities of gruel and whey, with a little wine occasionally, having no intestinal discharge whatever but what was made through the wound in his belly. On the 18th, as his strength was much increased, and as the wounds were considerably contracted, and looked well, I ordered another injection to be administered gently, when, for the first time in eleven days, he had a natural ftool. From this time he had no farther difcharge of fæces through his wound; his excretions became as regular and as natural as ever they were; his wounds fuppurated and healed kindly; his ftrength returned; and he was exchanged nearly as well as ever on the thirtieth.

That the ball had paffed through the colon is obvious, from the difcharge of perfect fæces and of the injection adminiftered, *ab ano*. That his life depended upon our not meddling with the wound, and upon keeping him quiet and eafy, is alfo plain; as the leaft removal of the orifice in the inteftine from the orifice through the abdomen, which were fo happily oppofed to each other, muft have been attended with a fatal difcharge of the fæces into the abdomen. That the diaphragm and lungs were perforated is plain, from the courfe of the ball, and his profufe hæmoptoe. That furgeons may be too officious as well as too tardy; and that where they are not certain of the utility of their operations they had better leave even the moft defperate diforders to the management of nature, ever provident, and generally adequate, are points remarkably enforced in this particular cafe.

